# Mesenchymal stem cells alleviate experimental cerebral malaria disease severity by inducing RoRγt^+^ Foxp3^+^ T regulatory (Tr 17) cells and modulating the dysregulated Th17/Treg axis

**DOI:** 10.1038/s41420-025-02900-3

**Published:** 2026-01-30

**Authors:** Indu Sharma, Reva Sharan Thakur, Amrendra Chaudhary, Rubika Chauhan, Kuldeep Singh, Srikanth Sadhu, Amit Awasthi, Jyoti Das

**Affiliations:** 1 Academy of Scientific and Innovative Research (AcSIR), Ghaziabad, Uttar Pradesh India; 2 Division of Immunology, National Institute of Malaria Research, Ghaziabad, Uttar Pradesh India; 3 Centre for Immunobiology and Immunotherapy, Translational Health Science and Technology Institute, NCR-Biotech Science Cluster, 3rd Milestone, Faridabad, India

**Keywords:** Cell death and immune response, Mesenchymal stem cells

## Abstract

Cerebral malaria (CM) is associated with dysregulated immune response against the blood stage of malaria parasite that often leads to serious organ damage, ultimately causing fatal pathological complications. Conventional treatments, although effective in controlling the parasite, often fail to address the severe immunopathology associated with the disease. Herein, we investigated the therapeutic potential of Mesenchymal stem cells (MSCs) in managing the excess proinflammatory response and maintaining immune homeostasis in *Plasmodium berghei* ANKA (PbA) infected C57BL/6 mice, an experimental cerebral malaria (ECM) disease model. Parasitemia and survival were monitored regularly, along with the neurological complications associated with the disease. Immunophenotyping, along with programmed cell death analysis of splenocytes, was also done via flow cytometry, and cytokine levels were analyzed at different time points in serum, as well as spleen, through bioplex assay and qRT-PCR. It was found that MSC effectively reduced parasitemia, increased survival, and decreased hemozoin accumulation in spleens of PbA-infected mice, along with improving brain pathology by preventing vascular leakage and protecting the blood–brain barrier (BBB). MSCs not only rescued the lymphocytes from apoptosis by downregulating PD-1/PD-L1 and ROS levels but also effectively modulated the Th17/Treg imbalance and maintained immune homeostasis by downregulating Interleukin-6 (IL-6) and Interleukin-17 (IL-17) cytokines and upregulating Interleukin-10 (IL-10) cytokine in infected mice. For the first time, we reported that MSCs were able to induce a dual phenotype effector Treg cell subset (Tr17), which are known to express both RoRγt and Foxp3 transcription factors, which were highly suppressive against pathogenic Th17 cells as they significantly downregulated IL-17 expression in Th17 cells. In conclusion, our findings offer insight into how the infusion of MSCs reduces the severity of experimental CM by modulating Th17/Treg balance and inducing Tr17 effector Treg response against Th17 cells. Thus, MSCs could potentially be used as an adjunct therapy for addressing the immunopathological complications of CM.

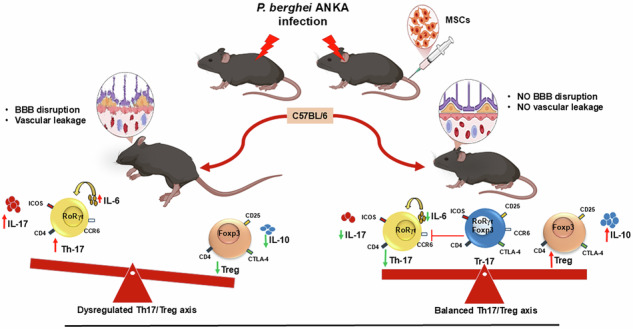

## Background

CM is a life-threatening neurological complication of *Plasmodium falciparum* infection and remains a major cause of mortality and long-term neurological sequelae, especially in young children [1]. An estimated 263 million malaria cases were reported worldwide in 2023, increasing by 11 million from the year 2022. In contrast to this, the death toll decreased from 622,000 in 2022 to 597,000 in 2023 [2]. CM in both humans and murine models is characterized by excessive immune activation, blood–brain barrier (BBB) disruption that ultimately leads to neuroinflammation, causing neurological damage [3, 4]. CM immunopathogenesis involves the dysregulation of CD4⁺ T cell subsets, particularly excess Th1 and Th17 responses that lead to higher production of proinflammatory cytokines such as IFN-γ and IL-17 [5]. Th17 cells are mainly characterized by the expression of RORγt transcription factor and the secretion of IL-17 cytokine on activation [6], which is known to promote inflammation, recruit neutrophils, and contribute to tissue damage, including BBB compromise during ECM [7, 8]. This excess proinflammatory response is, in general, counterbalanced by regulatory T cells (Tregs), characterized by the expression of Foxp3 transcription factor [9]. These T cells are known to maintain immune homeostasis through the secretion of TGF-β and IL-10 cytokines, thus changing the microenvironment [10] or through direct cell-cell contact via inhibitory surface molecules such as Cytotoxic T lymphocyte associated protein-4 (CTLA-4), which directly inhibits effector cell function and prevents immunopathology [11]. There is a critical balance between the Th17 and Treg cells that decides the disease outcome. The polarization of the CD4^+^ T-cell population, mainly the increase in proinflammatory Th17 cells due to a strong IL-6 environment and a reduction in Treg cells, is characteristic of CM and directs the immune response toward pathogenic inflammation that worsens disease outcomes [12]. A novel subset of Treg cells was initially identified against gut microbiota that co-expresses both Foxp3 and RORγt transcription factors and was required to regulate certain T cell subsets in the gut [13]. This subset was later reported in peripheral lymph nodes by Kim et al. [14] and could release IL-17 cytokines on re-stimulation and hence named Tr17 cells [15]. These dual-phenotype cells have high expression of Treg-associated surface molecules and have increased suppressive activity against pathogenic effector cells, mainly antigen-specific Th17 cells [14]. The presence of Tr17 cells in CM has not been reported and raises an intriguing question about their role in modulating the host immune response.

Conventional antiparasitic treatments like artemisinin combined therapies (ACT) are mainly used to treat *P. falciparum* infections [16] that are capable of effectively reducing parasitemia but often fail to prevent or reverse CM-mediated severe immunopathology [17], thus highlighting the urgent need for adjunctive therapies that target host immune dysregulation. Stem cell therapy, taking the immunomodulatory and regenerative ability of major stem cells, can prove beneficial as an adjunct therapy for managing disease severity in CM. Mesenchymal Stem Cells are one such example of multipotent cells that are widely used for the treatment of various inflammatory [18] and neurodegenerative disorders [19] due to their hypoimmunogenic nature and ease of isolation [20]. Reports suggest that owing to their plastic nature, MSCs act as immunosuppressive under inflammatory conditions (high INF-γ, TNF-α, etc.) [21], which are often observed in CM. These immunosuppressive MSC can modulate both innate and acquired immune responses by secreting certain soluble factors or by direct cell-cell interactions with immune cells [22]. MSCs have been reported to impede effector T cell response [23] and enhance or recruit regulatory T cell phenotypes [24], thus maintaining immune homeostasis. These cells, hence, could prove beneficial in controlling the excess proinflammatory response seen in CM and maintaining the Th17/Treg balance, preventing or reversing harmful immune deviation such as that seen with the Th17 cell population.

The current study evaluates the therapeutic potential of MSCs in the PbA-induced ECM model in C57BL/6 mice. We focused on MSC-mediated modulation of Th17, Treg, and Tr17 subsets, assessing their frequency, function, and associated cytokine profiles in spleen and lymph nodes. Our findings shed light on how MSCs influence the Th17/Treg/Tr17 axis, offering novel insights into their potential role as a host-directed therapy in the management of CM.

## Experimental procedure

### Experimental animals

Female C57BL/6 mice, aged 5–6 weeks and weighing 20–24 g, were acquired from the Animal Care Facility at the National Brain Research Centre (NBRC), Manesar. The mice were housed in polypropylene cages, with five mice per cage, and maintained under standardized environmental conditions of 24 ± 1°C temperature and 55 ± 10% humidity at the animal facility of the National Institute of Malaria Research (NIMR), New Delhi. They were provided with filtered water and a standard pellet diet ad libitum. All experimental procedures were conducted in accordance with the guidelines approved by the Institutional Animal Ethics Committee of NIMR, under approval number IAEC/NIMR/2022-2/04 and NIMR/IAEC/ 2024_2/1.

Foxp3 reporter mice (**C57BL/6-Foxp3**^**tm1Flv**^**/J-008374**) were purchased from The Jackson Laboratory, USA, and maintained under standard environmental conditions at the animal facility of the Translational Health Science and Technology Institute (THSTI), Haryana. All experimental procedures were conducted in accordance with the guidelines approved by the Institutional Animal Ethics Committee of THSTI with approval number IAEC/THSTI/404.

Animals were included in the study based on their age and body weight, with inclusion criteria designed to ensure that the average weight was comparable across all experimental groups. Animals were randomly assigned to the experimental groups to minimize selection bias and ensure comparable baseline characteristics across groups.

### *Plasmodium berghei* ANKA (PbA) Infection

The mice were infected following the procedure outlined by Reva et al. [25]. PbA strains, sourced from the BEI resources, were maintained as frozen stocks in liquid nitrogen vials. In brief, all experimental mice were intraperitoneally (i.p.) infected with 1 × 10^5^ PbA-infected red blood cells (iRBCs) suspended in 0.2 ml of phosphate-buffered saline (PBS), which were obtained via cardiac puncture from homologous mice that had previously been injected with the frozen PbA stock solution. During the progression of the disease, mice were monitored for parameters including weight loss, survival, blood parasitemia, and clinical signs of ECM. Infected (*n* = 5) and treated animals (*n* = 5) were evaluated daily, with the time of death recorded immediately upon occurrence. Behavioral alterations, such as ataxia and convulsions, were considered established humane endpoints to minimize animal suffering.

Parasitemia levels (percentage of parasitized red blood cells, pRBCs) were monitored daily by microscopic examination of Giemsa-stained (Sigma-Aldrich, Burlington, Massachusetts, USA) thin blood smears obtained from the tail vein of the mice. The parasitemia was calculated using the following formula: [(number of pRBCs)/(total number of RBCs counted)] × 100. The experiment outcomes were observed by two different investigators separately. The investigators were blinded to the experimental groups during analysis.

### Mesenchymal stem cell (MSC) treatment

To assess the potential therapeutic effect of MSCs in ECM, mice were randomly assigned to three groups: an uninfected control group (*n* = 5), a PbA-infected group (*n* = 5), and a PbA-infected group (*n* = 5) treated with MSCs. MSCs were isolated from PbA-infected mice at 7 days post-infection (DPI). Mice were euthanized, and splenocytes were harvested from the spleens of infected animals. MSCs were then sorted from the total splenocyte population using negative selection, achieved by depleting lineage-positive (lin+) cells with cell-specific beads from Miltenyi Biotec (Bergisch Gladbach, Germany). A total of 3–5 × 10^6^ MSCs in 100 µl of PBS (Sigma Aldrich) were adoptively transferred via tail vein (intravenous) injection into syngeneic mice infected with PbA-ANKA on the day of infection. Mice in the control and PbA-infected groups received 0.1 ml of PBS solution at the same time. MSCs were cultured in RPMI Media supplemented with FBS at 37 °C, their capacity to differentiation into adipocytes was checked by culturing them with adipogenesis inducing media containing 5 mM dexamethasone, 0.5 M 3-isobutyl-1-methylxanthine, 10 mg/ml insulin and 10 mM indomethacin for 6 days and then cultured with maintenance media containing 10 mg/ml insulin for 21 days by changing the media every 2-3 days and later staining the cells with Oil red O to check lipid accumulation in cells. The isolated MSCs were also characterized through characteristic markers via flow cytometry after staining with the following antibodies:

**Table:** 

Antibodies	Clone	Company
CD11b PE	M1- 70	BD Biosciences, USA
CD90 BV421	OX-7	BD Biosciences, USA
CD45 APC	30-F11	BD Biosciences, USA
SCa-1 BV421	D7	BD Biosciences, USA
CD29 PE	HM β1-1	BD Biosciences, USA
CD105 APC	MJ7/18	BioLegend, California, USA

### Isolation of splenocytes

Animals were euthanized at 1-, 3-, and 7-DPI, and spleens were harvested in cold RPMI 1640 medium. Single-cell suspensions were generated by mechanically disintegrating the spleens of uninfected (*n* = 3), PbA-infected (*n* = 3), and PbA+ MSC-infused mice (*n* = 3). Red blood cells (RBCs) were lysed using RBC lysis buffer (0.15 M NH4Cl, 10 mM KHCO3, 0.1 mM Na2EDTA), and the resulting cells were filtered with 70 μm nylon cell strainers and washed with PBS before being resuspended in RPMI 1640 medium (Thermo Fisher Scientific) supplemented with 10% fetal bovine serum (FBS, Thermo Fisher scientific) and 100 IU/ml penicillin–streptomycin (Thermo Fisher Scientific) for further investigations.

### Flow cytometric analysis

Immunophenotyping of splenocytes was done by Flow cytometry at 1-, 3-, and 7-DPI. For intracellular staining, cells were restimulated with 1 μg/ml anti-CD3 and 2 μg/ml of anti-CD28 in RPMI 1640 medium (Thermo Fisher Scientific) supplemented with 10% FBS and 100 IU/ml penicillin–streptomycin. Golgi Plug protein transport inhibitor containing Monensin (BD Biosciences, Franklin Lakes, New Jersey, USA) was added after 6 h of incubation at a final concentration of 1 μg/ml to inhibit cytokine secretion. After 4 h, cells were harvested and re-suspended in 30 μl of FACS buffer (PBS, 2% FBS) and surface-stained at 4 °C with anti-mouse conjugated antibodies. Cells were washed twice with staining buffer and fixed with 1% paraformaldehyde. For intracellular staining, cells were washed twice with PBS and re-suspended in a permeabilization buffer (Cytofix/Cytoperm kit; BD), and stained with fluorescent conjugated antibodies. Fluorescence intensity was measured by flow cytometry (FACS Fortessa). Stained cells were acquired with a FACS Fortessa (BD) and analyzed by FlowJo (Tree Star) software. The following conjugated antibodies were used for staining:

**Table:** 

Antibodies	Clone	Company
CD4 perCP	GK1.5	BioLegend, USA
IL-6 PE	Cat-554401	BD Biosciences
IL-17 PE	Cat-550502	BD Biosciences
IL- 10 PE	JES 5-16E3	BioLegend,
Fox P3 APC	Flk	Invitrogen,CaliforniaUSA
RORγt Af700	IC9125N	R&D, Minnesota, USA
CC6R- BV421	29-2L17	BioLegend
CTLA (co 152) PE	VC10-4F10-11	BD Biosciences
ICOS PE	7E17G9	BioLegend
CD25- FITC	3C7	BioLegend
TGFβ – PE	TW7-16 B4	BioLegend

### ROS estimation with DCFH-DA staining

The level of ROS in splenocytes was estimated by staining the cells with 20 µM DCFH-DA stain. The cells were incubated at 37 °C in the dark for 40 min and washed with 1× PBS. Stained cells were acquired with a FACS Fortessa (BD Biosciences) and analyzed by FlowJo (Tree Star) software.

### Estimation of cell death and apoptosis

Splenocytes were isolated on 7 DPI, briefly washed with PBS, suspended in binding buffer, and stained with Annexin-V and PI (Annexin V apoptosis detection kit, Abkine, China) according to the manufacturer’s protocol. The cells were incubated at room temperature in the dark for 20 min and washed with 1× PBS. Stained cells were acquired with a FACS Fortessa (BD Biosciences) and analyzed by FlowJo (Tree Star) software.

### Evans blue dye perfusion for evaluation of BBB leakage

BBB integrity was assessed by measuring Evans blue dye extravasation, as described by Kim et al. (2014) [25]. Briefly, on day 7 post-infection, mice were intravenously injected with 100 µl of 1% Evans blue dye (Sigma-Aldrich) prepared in sterile PBS. Following this, the mice were euthanized and perfused with 20 ml of PBS to remove circulating dye. The brains were then harvested, photographed, and immersed in formamide for 48 h to extract the Evans blue dye. The concentration of Evans blue dye in the formamide was measured at 620 nm using a plate reader and quantified by comparison to a standard dye curve. Data were expressed as micrograms of dye extravasated per milligram of brain tissue.

### RNA isolation and real-time PCR

Splenocytes were isolated on 1, 3, 7 DPI (*n* = 3) and 1 × 10^7^ cells were snap frozen after adding trizol reagent (BR Biochem, India). RNA was isolated from these splenocytes manually, and first-strand synthesis was carried out by using a high-capacity cDNA kit (Thermo Fisher Scientific). mRNA expression levels of specific genes were quantified by real-time PCR (Biorad CFX 96 real-time PCR, Hercules, California, USA) using SYBR master mix (iTaq universal SYBR Biorad). Thermocycler settings included an initial incubation of 5 min at 95 °C followed by a cycle of denaturation (10 s at 95 °C), annealing (10 s at 60 °C), and extension (10 s, 70 °C) repeated 40 times. Total mRNA levels were normalized across all samples using endogenous levels of beta-actin mRNA. Relative fold change was calculated using the delta delta Ct method. The primer sequence for different genes studied was as follows:

β-actin Forward 5′-GATCATGTTTGAGACCTTCAACACC-3′

β-actin Reverse 5′-CGTGAGGGAGAGCATAGCCC-3′,

IL-17-Forward 5′-CTCCAGAAGGCCCTCAGACTAC-3′

IL-17-Reverse 5′-AGCTTTCCCTCCGCATTGACACAG-3′

IL-10-Forward 5′-TGCTAACCGACTCCTTAATGCAGGAC 3′

IL-10-Reverse 5′-CCTTGATTTCTGGGCCATGCTTCTC-3′

IL-6-Forward 5′-CCGGAGAGGAGACTTCACAG-3′

IL-6-Reverse 5′-TGGTCTTGGTCCTTAGCCAC-3′

### Histological analysis by H&E staining

Spleen was isolated from control, PbA-infected, and PbA + MSC infused mice on 1, 3, and 7 DPI (*n* = 3) and fixed in 10% formalin (Sigma Aldrich). The fixed tissue was embedded in paraffin, and 3–4 μm sections were cut onto the slides. These sections were stained with hematoxylin and eosin and viewed under a 100× objective in a light microscope to study the hemozoin accumulations in all the groups. Brains were also removed on 7 DPI from all the groups and stained with hematoxylin and eosin in a similar manner. Brain sections were viewed under 20× and 100× to study CM-mediated structural changes and any changes in MSC infusion.

### Cytokine analysis

Blood was collected from all the animals on 1, 3, and 7 DPI (*n* = 3) and serum was isolated and stored in −80 °C. The samples were then analyzed for cytokine levels by multiplexed bead array immunoassay using Luminex technology (Bio-Plex; Bio-Rad) according to the manufacturer’s protocol.

### In vitro suppression assay

CD4^+^ T cells were isolated from spleens of PbA-infected and PbA-infected MSC-infused Foxp3 reporter mice (*n* = 3) on 7-DPI by positive selection using cell-specific beads from Miltenyi Biotec (Bergisch Gladbach, Germany). CD4^+^CCR6^+^ICOS^hi^RFP^−^ Th17 cells and CD4^+^CCR6^+^ICOS^hi^RFP^+^ Tr17 cells were further FACS-sorted from these CD4^+^ T cells and co-cultured on anti-CD3e and anti-CD28-coated plates in the ratio (Tr17:Th17) of 1:1 and 1:5. After 72 h of co-culture, supernatant was collected to check the level of cytokines, and cells were harvested for immunophenotyping.

### Estimation of MSC-derived NO and PGE2

Isolated MSCs from spleen were cultured in RPMI-1640 supplemented with 10% FBS till confluency and were then seeded in 24-well plates. After 24 h, spent media was collected for the determination of NO levels by Griess reagent and PGE2 levels through ELISA (Caymen Chemical, Michigan, USA).

### IL-10 neutralization and inhibition of COX-2 and iNOS

To inhibit the effect of IL-10 in vivo, we injected the PbA-infected mice with IL-10-neutralizing antibody (JES3-9D7, eBiosciences). The mice were infected with PbA and divided randomly into three groups: PbA infected, PbA infected + anti-IL-10, PbA infected + IgG control group (*n* = 5). IL-10 was injected intravenously with concentrations of 25 ug, 50 ug, 70 ug, and 100 ug per mouse on 2, 3, 4, 5 DPI. Corresponding concentrations of IgG were given in the PbA-infected + IgG control group.

To inhibit expression of COX 2 and iNOS, mice (*n* = 4) were orally fed with Celebrex in PBS (10 mg/ kg body weight) twice daily till 7DPI, and their parasitaemia and survival were monitored.

### Statistical analysis

All data were expressed as the mean ± standard error mean (SEM). Statistical analyses were performed using a two-sided statistical test, which was either one-way ANOVA followed by post-test Tukey–Kramer or two-way ANOVA followed by Bonferroni post-test, which checks the variance in comparison groups before performing the analysis. The variance was similar in the comparison groups. Mice were randomly divided into groups of five animals (per group), and all data are representative of at least two independent experiments. Analyses were conducted with the GraphPad Prism Software, and significant differences were defined with *p* values less than 0.05.

## Results

### MSCs improve the pathophysiology of CM, protect the brain from vascular leakage, and maintain the integrity of the BBB in PbA-infected C57BL/6 mice

**Fig. 1:**
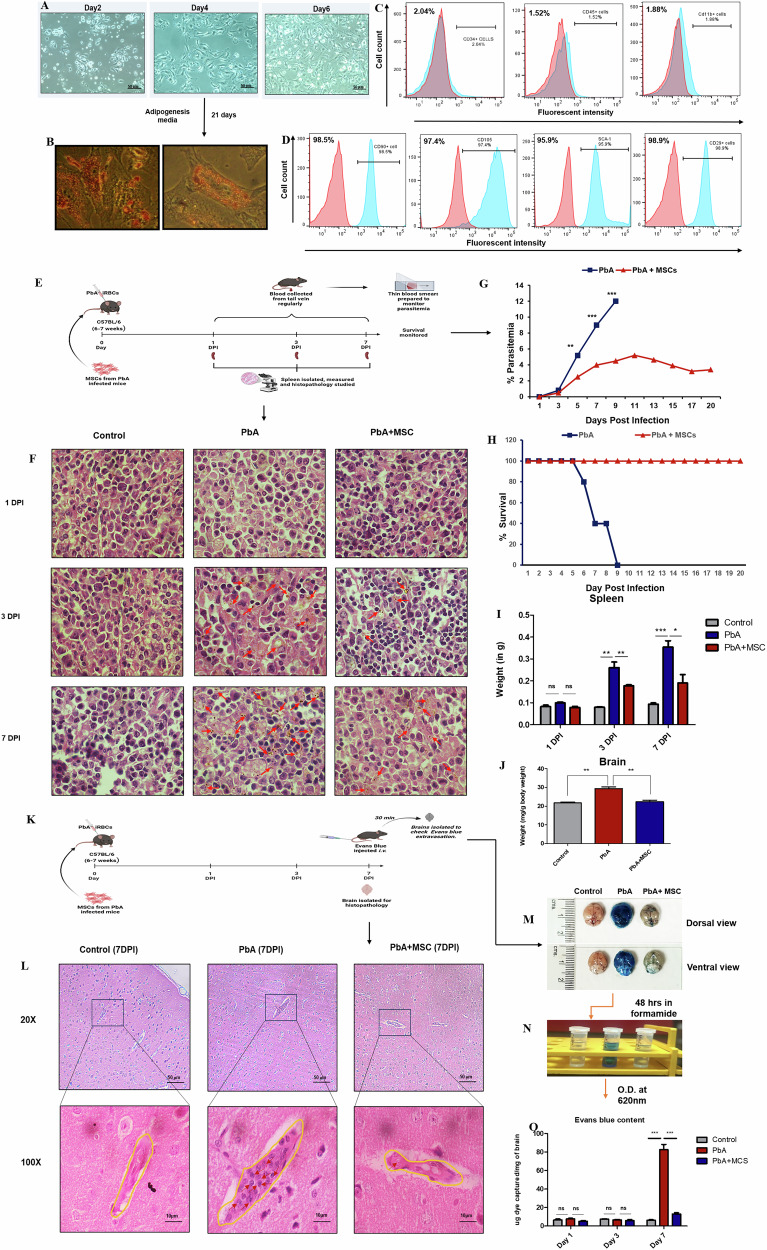
MSC infusion improves the pathophysiology of CM and maintains BBB integrity in PbA-infected. Parasitemia was monitored regularly from thin blood smears of PbA-infected (*n* = 5) and MSC (*n* = 5) mice, and spleens were collected on 1-, 3-, and 7-DPI for histological analysis. Mice were injected with 1% Evans blue on 7 DPI, after which brains were isolated from all three groups and analyzed for BBB rupture. **A** Representative microphotographs of in vitro cultured MSCs at 20× magnification on Day 2, 4, and 6. **B** Representative microphotographs of Oil red O-stained adipocytes differentiated from MSCs. **C**, **D** Characterization of MSCs. **E** Diagram for an in-vivo experimental setup for analyzing pathophysiology. **F** Representative microphotographs of spleen at 100× magnification showing hemozoin content at 1-, 3-, and 7- DPI. Quantification of dye captured by the brain parenchyma. **G** Parasitemia curve showing difference in increase of parasite load in PbA-infected and MSC-infused PbA-infected mice (*n* = 5). **H** Survival curve showing increased survival in MSC-infused PbA-infected mice (*n* = 5). **I** Bar graph showing wet weights of spleen in Control, PbA infected, and MSC infused PbA infected mice at 1-, 3-, and 7- DPI, respectively. **J** Bar graph showing wet weights of brain normalized to body weight in Control, PbA infected, and MSC infused PbA infected mice (n = 3) at 1-, 3-, and 7- DPI, respectively. **K** Diagram for in vivo experimental setup for analyzing BBB rupture. **L** Representative microphotographs of the cerebral cortex at 20 and 100× magnification showing dilation and obstruction in blood vessels owing to the PbA infection. **M** Representative photographs showing qualitative analysis of whole brains of Control, PbA-infected, and MSC-infused PbA-infected mice after Evans blue administration. **N** Photographs showing the extraction of captured by the brain in formamide. **O** Bar graph showing quantification of dye captured by brain parenchyma. The data shown is representative of two independent experiments. Data are expressed as mean ± SEM. One-way ANOVA, followed by post hoc Tukey test, was used to determine statistical significance. ^*^p < 0.05, ^**^p < 0.01, ^***^p < 0.001, ns non-significant. Scale bar = 10 µm for 100× and 50 µm for 20× magnification.

The effect of MSC infusion on the progression of CM was assessed in PbA-infected C57BL/6 mice. MSCs were isolated from spleens of PbA-infected mice and were cultured in vitro (Fig. 1A) to determine their differentiation potential by culturing them in adipocyte-differentiating media and then staining these cells for identification of lipid globules a characteristic feature of mature adipocytes (Fig. 1B). A part of isolated cells was also characterized with specific markers via flow cytometry and were positive for several stromal markers like CD29, CD90, CD105 and Sca-1 (Fig. 1D), on the other hand these cells were negative for hematopoietic markers like CD11b, CD 45 and CD 34 (Fig. 1C). These isolated cells were therefore defined as MSCs as per the International Society for Cellular Therapy (ISCT). MSCs isolated from spleen also produced copious amounts of NO, as well as PGE2, which was much higher than the MSCs isolated from bone marrow when cultured in vitro (Supplementary Fig. 1D, E)*.*

MSCs were adoptively transferred on the same day as infection, and parasitemia, survival, and neurological symptoms were monitored. Consistent with previous studies (Reva et al. 2022), parasitemia in PbA-infected mice increased rapidly after DPI, peaking at 7 DPI, with about 60% mortality observed by this time and 100% mortality by 9 DPI. However, MSC infusion in infected mice resulted in a slower increase in parasitemia and a reduced parasite load, leading to improved survival, with no mortality being observed till 20 DPI (Fig. 1G, H). MSC infusion also alleviated splenomegaly observed in PbA-infected mice, as the spleens of MSC-infused mice were significantly smaller compared to those of the PbA-infected mice (Fig. 1I). Histological analysis of H&E-stained slides of spleen revealed increased accumulation of hemozoin content at 3 DPI and 7 DPI in PbA-infected mice. This accumulation of hemozoin was altogether reduced in MSC-treated mice (Fig. 1F). Moreover, MSC treatment significantly alleviated neurological symptoms, such as ataxia and convulsions, which were less severe in the MSC-infused group compared to the PbA-infected mice without MSC treatment. This improvement in neurological function was further supported by a lower wet weight of brains isolated from the MSC-infused group, as compared to the PbA-infected group (Fig. 1J), suggesting a decrease in brain edema and inflammation. These findings suggest that MSC infusion has a protective effect against both parasitemia progression and neurological damage, enhancing survival in a murine model of CM.

**Fig. 2:**
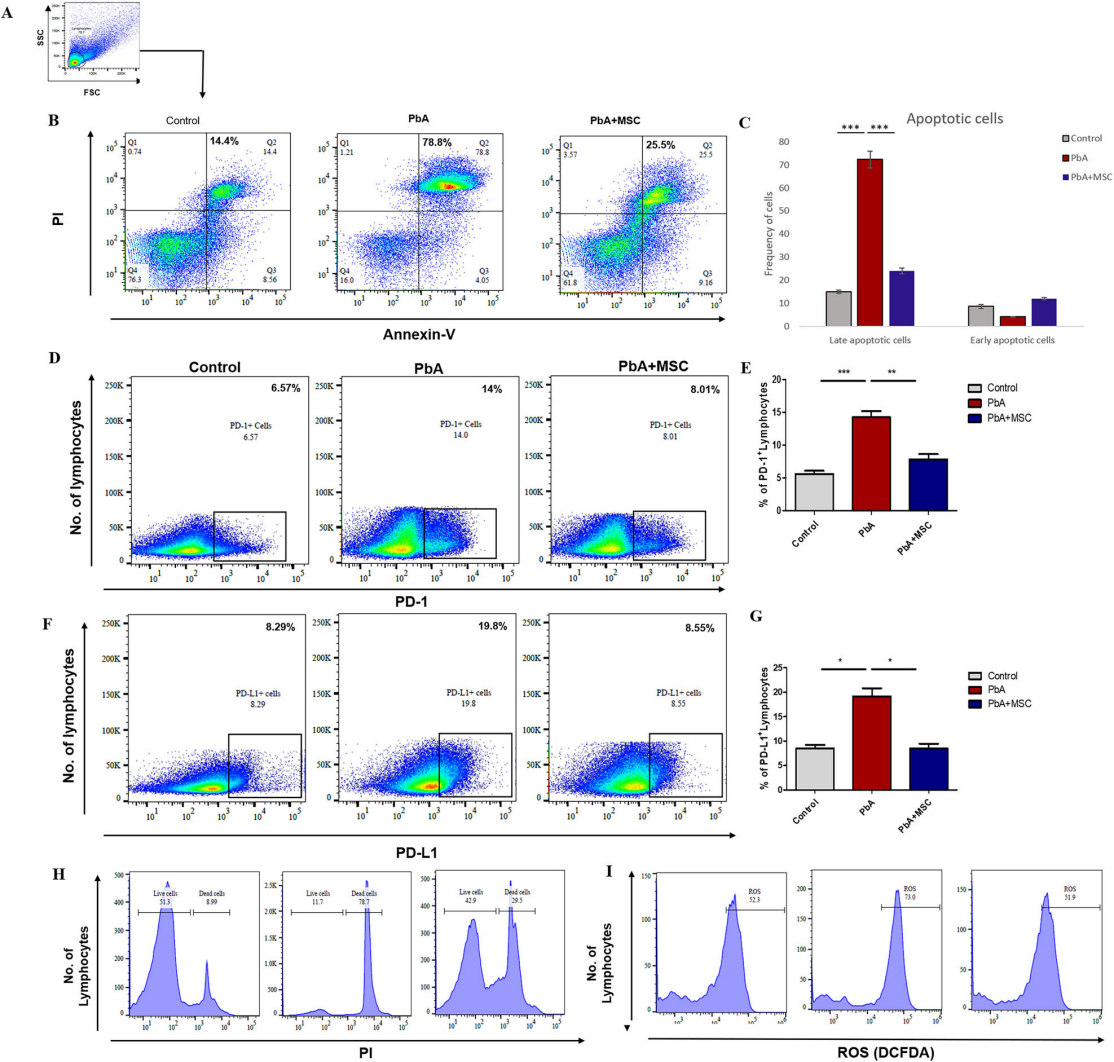
MSC infusion decreases the level of PD-1 and ROS levels in lymphocytes, thereby decreasing their apoptosis. Mice were infected with PbA, infused with MSCs, and splenocytes were isolated on 7 DPI, after which they were stained with Annexin-V and PI for apoptosis and with DC-DFDA for ROS levels. **A** Representative FACS plot showing the gating strategy for the identification of lymphocytes. **B**, **C** Representative flow plots and bar graphs showing frequency of apoptotic cells in spleen of control (*n* = 3), PbA infected (*n* = 3), and MSC infused PbA infected mice (*n* = 3) on 7 DPI, respectively. **D**–**G** Representative flow plots and bar graphs showing frequency of PD-1^+^ and PD-L1^+^ lymphocytes in spleen of control (*n* = 3), PbA infected (*n* = 3) and MSC infused PbA infected mice (*n* = 3) on 7 DPI, respectively. **H** Histograms showing the number of live and dead lymphocytes in control (*n* = 3), PbA-infected (*n* = 3), and MSC-infused PbA-infected mice (*n* = 3) on 7 DPI. **I** Histograms showing ROS levels in lymphocytes of control (*n* = 3), PbA infected (*n* = 3) and MSC infused PbA infected mice (*n* = 3) on 7 DPI. The data shown is representative of two independent experiments. Data are expressed as mean ± SEM. One-way ANOVA, followed by post hoc Tukey test, was used to determine statistical significance. ^*^p < 0.05, ^**^p < 0.01, ^***^p < 0.001.

A prominent characteristic observed in both human and experimental CM is the infiltration of immune cells into the brain and the disruption of the BBB [26], resulting in vascular leakage and subsequent neurological complications [27]. To evaluate the impact of MSCs on BBB integrity, animals were administered Evans blue dye, which binds to albumin in the blood and only permeates into the brain parenchyma in the event of vascular leakage. As depicted in Fig. 1M, MSC-treated PbA-infected mice exhibited minimal Evans blue staining, indicating lesser dye extravasation into the brain compared to PbA-infected mice without MSC treatment. The Evans blue dye that entered the brain parenchyma was extracted using formamide (Fig. 1N), and subsequent quantification revealed a significant increase in BBB permeability in PbA-infected mice, with a 70% (*p* < 0.001) increase in Evans blue extravasation compared to uninfected controls. In contrast, MSC treatment significantly attenuated Evans blue dye extravasation, showing only a 15% (*p* < 0.001) increase in comparison to the uninfected group (Fig. 10). Histological analysis of the cerebral cortex from PbA-infected mice shows dilated vessels along with obstruction due to the accumulation of cells in the brain vasculature. Interestingly, MSCs reduced this PbA-mediated dilation and blood vessel obstruction, with fewer cells being recruited to the brain vasculature (Fig. 2L). These results suggest that MSCs confer protection against BBB disruption induced by CM, a hallmark feature observed in both human and experimental models of the disease.

### MSCs rescue apoptotic lymphocytes by downregulating PD-1 expression and cellular ROS levels

**Fig. 3:**
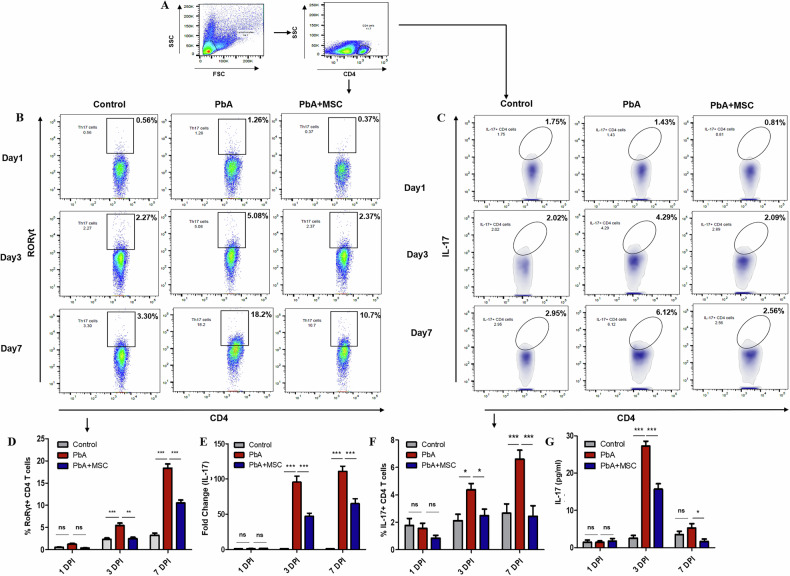
Decreased Th17 response in MSC-treated murine models of CM. PbA-infected mice were infused with MSCs, and splenocytes were isolated for characterization of RORγt^+^ CD4^+^ T cells and IL17-secreting CD4^+^ T cells s through flow cytometry. **A** Representative FACS plot showing the gating strategy for the identification of Th17 cells. **B** Representative flow plots and **D** bar graphs showing frequency of Th17 cells in spleen of control, PbA-infected and MSC-infused PbA-infected mice (n = 3) on 1, 3 and 7 DPI, respectively. **C** Representative flow plots and **F** bar graphs showing frequency of IL17 secreting CD4^+^ T cells s cells in spleen of control, PbA infected and MSC infused PbA infected mice (*n* = 3) on 1, 3, and 7 DPI, respectively, **E** mRNA expression of IL-17 gene in splenocytes of control, PbA infected and MSC infused PbA infected mice (*n* = 3) on 1, 3 and 7 DPI, and **G** level of IL-17 cytokine in serum of control, PbA infected and MSC infused PbA infected mice (*n* = 3) on 1, 3 and 7 DPI. The data shown is representative of two independent experiments. The data shown is representative of two independent experiments. Data are expressed as mean ± SEM. Two-way ANOVA, followed by post hoc Bonferroni method, was used to determine statistical significance. ^*^p < 0.05, ^**^p < 0.01, ^***^p < 0.001, ns non-significant.

Extensive apoptosis of lymphocytes is seen in the spleen during malaria infection, which can alter the immune response [28]. Thus, we checked the effect of MSC infusion on cell death in PbA-infected mice. Staining of splenocytes with Annexin-V on 7 DPI revealed an increase (*p* < 0.001) in the number of lymphocytes in the late apoptotic phase in PbA-infected mice as compared to the control mice (Fig. 2B, C). Live-dead staining with PI also showed an increase in the number of dead lymphocytes in response to PbA infection (Fig. 2H). Interestingly, MSCs rescued the lymphocytes from PbA-mediated apoptosis as the number of late apoptotic and dead lymphocytes decreased dramatically (*p* < 0.001) in MSC-infused mice as compared to the PbA-infected mice. Next, we analyzed the expression of PD-1 and PD-L1 on lymphocytes, as it is known to cause lymphocyte exhaustion and apoptosis [29]. We found that the expression of both PD-1 and PD-L1 was significantly (*p* < 0.001 and *p* < 0.05) upregulated on splenic lymphocytes in PbA infected mice whereas this was reversed in MSC infused PbA infected mice as the PD-1 and PD-L1 levels on lymphocytes show significant downregulation (*p* < 0.001 and *p* < 0.05) as compared to the only infected mice (Fig. 2D–G). We further checked the oxidative stress in lymphocytes since extensive ROS generation can cause DNA fragmentation, cellular damage, and may activate apoptosis in cells [30]. PbA infection increased the oxidative stress in lymphocytes, as evident by increased ROS production, which was significantly downregulated in MSC-infused mice (Fig. 3I). These results suggest that MSCs were able to downregulate PD-1 and ROS levels in lymphocytes, ultimately rescuing them from apoptosis.

### MSCs attenuate Th17 cell-mediated inflammatory responses in experimental cerebral malaria (ECM)

Th17 cells, characterized by the expression of the transcription factor RORγt and production of the proinflammatory cytokine IL-17, play a crucial role in mediating host immune responses. However, their excessive activation has been implicated in the pathogenesis of ECM. To check the effect of MSC on Th17 response, RORγt^+^ CD4^+^ T cells were gated after gating lymphocytes in a scatter plot. The frequency of RORγt^+^ CD4^+^ T cells increased rapidly as the disease progressed, the increase being most significant on 3 DPI (*p* < 0.001) and 7 DPI (*p* < 0.001) as compared to the control group (Fig. 3B, D). This increase in the population of Th17 cells was consistent with increasing parasitemia and disease severity. However, MSCs infusion into PbA-infected mice significantly decreased the frequency of RORγt^+^ CD4^+^ T cells, thus managing the excessive Th17-mediated proinflammatory response (Fig. 3B, D). We further analyzed IL-17-secreting CD4^+^ T cells in the spleen, and consistent with the above results, their IL-17 + CD4^+^ T cells population was higher in PbA-infected mice as compared to control mice. However, this increase was ameliorated in mice that received MSCs, and the population of IL-17-secreting CD4^+^ T cells was almost comparable to that seen in control mice (Fig. 3C, F). Our results were confirmed through gene expression studies in splenocytes as mRNA expression for the gene encoding IL-17 was significantly downregulated as only 40-to-50-fold (*p* < 0.001) change was observed (*p* < 0.001) on treatment with MSCs whereas in mice with only PbA infection the fold change was quite higher about 100 to 110-fold (*p* < 0.001) as compared to control mice (Fig. 3E). Finally, we quantified IL-17 characteristic cytokine released by Th17 cells on activation in response to infection, in the serum of control, PbA-infected, and MSC-infused mice. Supporting our previous results, we found that MSCs significantly reduced the level of IL-17 cytokine on 3 and 7 DPI as compared with the PbA-infected mice, which show increased levels of the cytokine (*p* < 0.001 on 3DPI and *p* > 0.05 on 7 DPI, Fig. 3G).

### MSCs enhance regulatory T cell (Tregs) response in PbA-Infected Mice

**Fig. 4:**
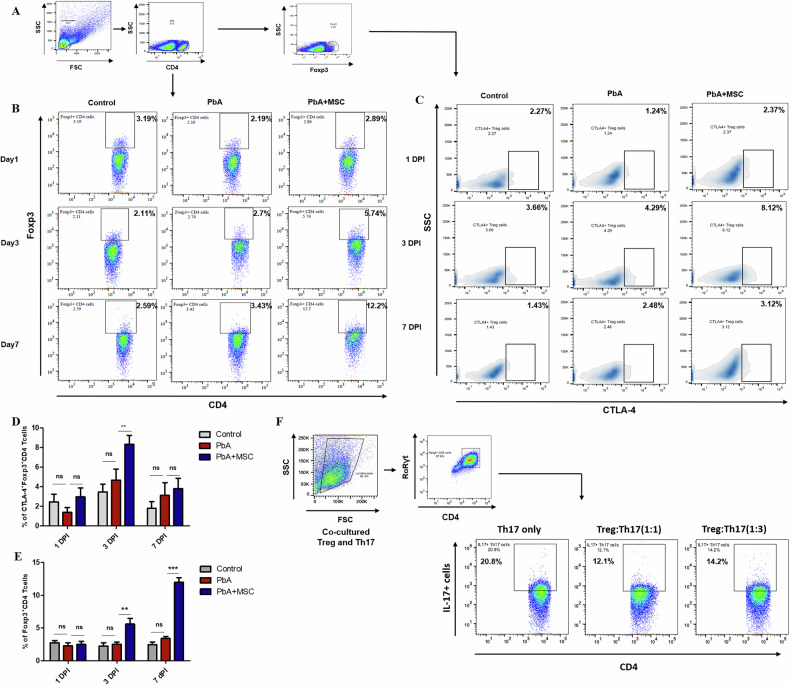
Increased Treg cell response in MSC treated CM disease model. PbA-infected mice were infused with MSCs, and splenocytes were isolated for characterization of Foxp3^+^ CD4^+^ T cells and CTLA-4^+^ Treg cells through flow cytometry. **A** Representative FACS plot showing the gating strategy for the identification of Treg cells. **B** Representative flow plots and **D** bar graphs showing frequency of Foxp3^+^ CD4^+^ T cells in spleen of control, PbA infected and MSC infused PbA infected mice (*n* = 3) on 1, 3 and 7 DPI, respectively. **C** Representative flow plots and **E** bar graphs showing frequency of CTLA-4^+^ Treg cells in spleen of control, PbA-infected and MSC-infused PbA-infected mice (*n* = 3) on 1, 3 and 7 DPI, respectively. **F** Representative flow plots showing frequency of IL-17^+^ Th17 cells in in vitro *culture* of Th17 only, Treg:Th17 (1:1), and Treg:Th17 (1:3) (*n* = 3), respectively. The data shown is representative of two independent experiments. The data shown is representative of two independent experiments. Data are expressed as mean ± SEM. Two-way ANOVA, followed by post hoc Bonferroni method, was used to determine statistical significance. ^*^p < 0.05, ^**^p < 0.01, ^***^p < 0.001, ns non-significant.

Regulatory T cells (Tregs), characterized by expression of the Foxp3 transcription factor, are crucial in maintaining immune homeostasis by controlling the excess pro-inflammatory response that may contribute to disease pathogenesis. Therefore, we analyzed the effect of MSCs on Treg response in the spleen, as well as lymph nodes. We found that adoptive transfer of MSCs in PbA-infected mice resulted in a significant increase in the Treg cell population on the 3rd (*p* < 0.01), as well as the 7th (*p* < 0.001), as compared to the PbA-infected mice, which showed no change in frequency of Tregs over the course of infection (Fig. 4B, D). Next, we checked the expression of CTLA-4 on the Treg cell population in all the animal groups, as it has been attributed to the functional aspects of Treg cells and is known to regulate the Th17 cell population. Interestingly, we observed a steady increase in the CTLA-4+ Treg cell population in MSC-infused mice, the increase being most significant on 3 (*p* < 0.01) DPI (Fig. 4C, E). To further confirm the ability of Treg cells to suppress the functionality of Th17 cells suppression assay was done by co-culturing Foxp3^+^ CD4 + T cells sorted from the spleenocytes of MSC-infused Foxp3^RFP^ reporter mice with Foxp3^−^ ICOS^hi^ CCR6^+^ Th17 cells sorted from PbA-infected Foxp3^RFP^ reporter mice. The Treg cells isolated from the MSC-infused mice were able to suppress the IL-17 expression in Th17 cells from PbA-infected mice (Fig. 4F). These results suggest that MSCs significantly increase Treg response in CM, which can control the effector function of Th17 cells.

### MSCs maintain immune homeostasis by upregulating the anti-inflammatory IL-10 cytokine

**Fig. 5:**
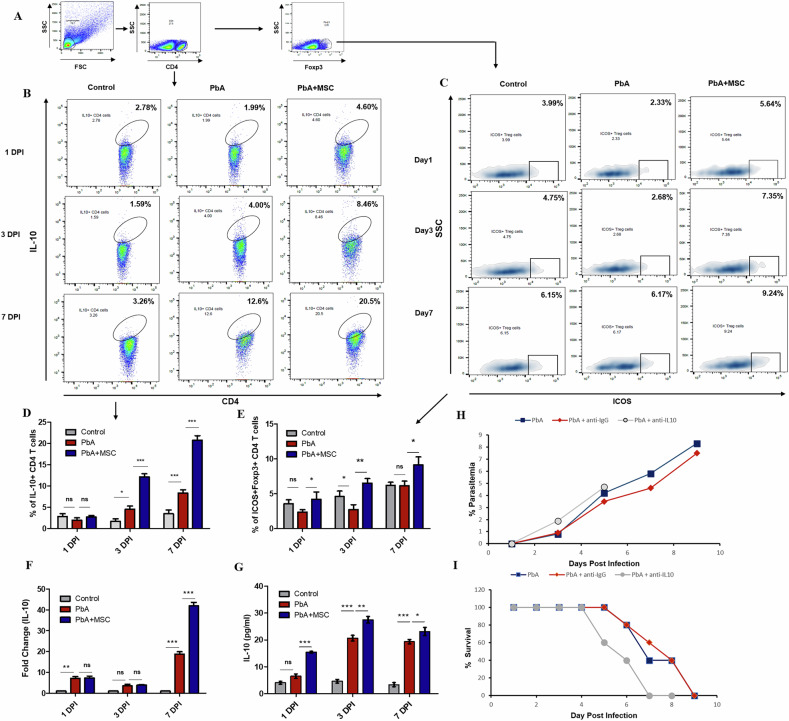
Elevated IL-10 levels on MSC infusion maintain immune homeostasis in PbA-infected mice. PbA-infected mice were infused with MSCs, and splenocytes were isolated for characterization of IL-10^+^ CD4^+^ T cells through flow cytometry. **A** Representative FACS plot showing the gating strategy for identification of IL-10^+^ CD4^+^ T cells. **B** Representative flow plots and **D** bar graphs showing frequency of IL-10^+^ CD4^+^ T cells in spleen of control, PbA infected, and MSC infused PbA infected mice (*n* = 3) on 1, 3, and 7 DPI, respectively. **C** Representative flow plots and **E** bar graphs showing frequency of ICOS^+^ Treg cells in spleen of control, PbA-infected, and MSC-infused PbA-infected mice (*n* = 3) on 1, 3, and 7 DPI, respectively. **F** mRNA expression of IL-10 gene in splenocytes of control, PbA-infected, and MSC-infused PbA-infected mice (*n* = 3) on 1, 3, and 7 DPI. **G** Level of IL-10 cytokine in serum of control, PbA-infected, and MSC-infused PbA-infected mice on 1, 3, and 7 DPI. The data shown is representative of two independent experiments. **H** Parasitemia curve showing in PbA-infected, PbA + anti-IgG, and PbA + anti-IL-10 infected mice (*n* = 5). **I** Survival curve showing decreased survival in PbA + anti-IL-10 infected mice (*n* = 5). The data shown is representative of two independent experiments. Data are expressed as mean ± SEM. Two-way ANOVA, followed by post hoc Bonferroni method, was used to determine statistical significance. ^*^p < 0.05, ^**^p < 0.01, ^***^p < 0.001, ns non-significant.

Apart from CTLA-4-mediated effector cell regulation, Treg cells can exert their regulatory function via other mechanisms, like the secretion of IL-10 cytokine, which plays a crucial role in limiting inflammation and maintaining immune homeostasis. We thus examined the IL-10 + CD4^+^ T cells in the control, PbA-infected, and MSC-infused groups. Consistent with the above results, the population of IL-10 secreting CD4^+^ T cells increased with infection in both PbA (*p* < 0.05 on 3 DPI and *p* < 0.001on 7 DPI) infected, as well as MSC-infused PbA infected mice (Fig. 5B, D). However, the increase was much more rapid and significant in MSC-infused mice as compared to PbA-infected mice (*p* < 0.001). Gene expression studies also revealed a significant upregulation of IL-10 mRNA expression in the splenocytes of MSC-treated mice on 7 DPI (*p* < 0.001, Fig. 5F). Serum cytokine analysis confirmed the significant upregulation in IL-10 cytokine levels in the serum of MSC-treated mice as compared to PbA-infected mice on 1 DPI (*p* < 0.001), 3 DPI (*p* < 0.01), and 7 DPI (*p* < 0.05), respectively (Fig. 5G). Next, we analyzed the ICOS^+^ Treg cells as they are known to secrete high levels of IL- 10 cytokines as compared to ICOS^−^ Treg cells [31] and found that the population of ICOS^+^ Treg cells was significantly higher in MSC-infused mice on 3 DPI (*p* < 0.01) and 7 DPI (*p* < 0.05) as compared to the PbA-infected mice (Fig. 5C, E). We further confirmed the protective role of IL-10 cytokine against cerebral malaria (CM) disease severity by neutralizing IL-10 cytokine by injecting neutralizing antibody in PbA-infected mice (Fig. 5F). We found that the neutralization of IL-10 in vivo resulted in increased lethality of PbA infection, as the survival of mice with anti-IL-10-infused PbA mice was lower compared to the PbA-infected mice, whereas there was no significant change in parasite load (Fig. 5H, I). In summary, these results indicate that MSCs might have the potential to increase the secretion of IL-10 by inducing the expression of ICOS on the surface of Treg cells, which reduces the susceptibility of mice to CM and thus maintains immune homeostasis.

### MSCs restore Th17/Treg balance in PbA-infected mice by modulating IL-6 and TGFβ cytokine levels

**Fig. 6:**
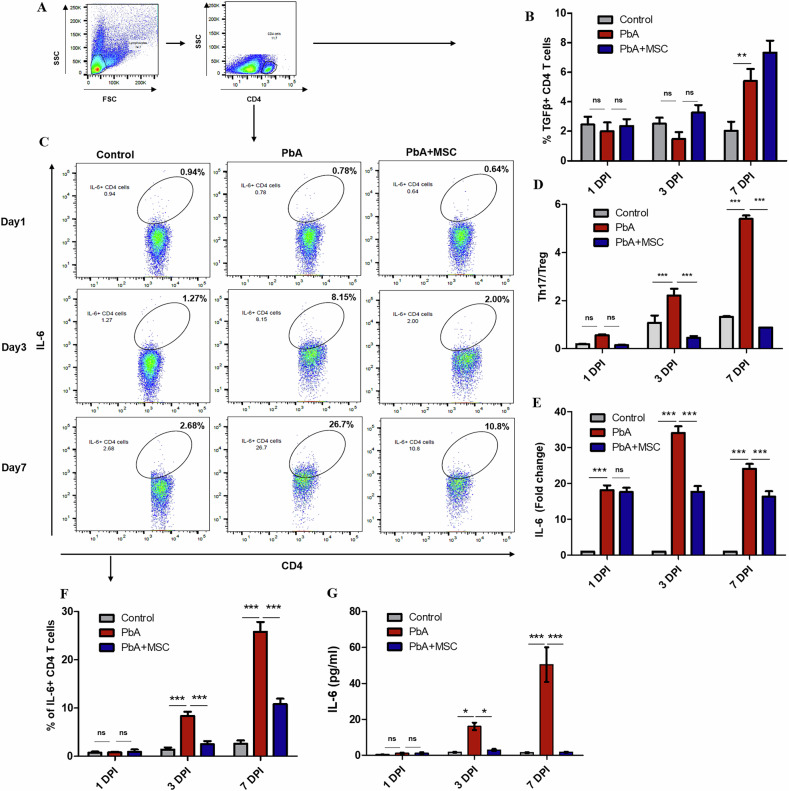
MSCs regulate the Th17/Treg balance via downregulation of IL-6 cytokine. PbA-infected mice were infused with MSCs, and splenocytes were isolated for characterization of IL-6^+^ CD4 + T cells s through flow cytometry. **A** Representative FACS plot showing the gating strategy for the identification of IL-6^+^ CD4^+^ T cells. **C** Representative flow plots and **F** bar graphs showing frequency of IL-6^+^ CD4^+^ T cells in spleen of control, PbA infected and MSC infused PbA infected mice (*n* = 3) on 1, 3 and 7 DPI, respectively, **B** bar graphs showing frequency of TGF-β^+^ CD4^+^ T cells in spleen of control, PbA infected and MSC infused PbA infected mice on 1, 3 and 7 DPI, respectively. **D** Bar graph representing the Th17/Treg ratio calculated from Th17 and Treg cell frequency. **E** Level of IL-6 cytokine in serum of control, PbA infected, and MSC-infused PbA-infected mice on 1, 3, and 7 DPI. **G** mRNA expression of IL-6 gene in splenocytes of control, PbA-infected, and MSC-infused PbA-infected mice on 1, 3, and 7 DPI. The data shown is representative of two independent experiments. Data are expressed as mean ± SEM. Two-way ANOVA, followed by post hoc Bonferroni method, was used to determine statistical significance. ^*^p < 0.05, ^**^p < 0.01, ^***^p < 0.001, ns non-significant.

Th17 and Treg cells share the same axis of development due to involvement of IL-6 and TGF-β cytokines, any change in these cytokines will bring about a change in the Th17 cell population, which will evidently change the Treg cell population dynamics and shift the Th17/Treg balance [32]. To check the effect of MSCs on Th17/Treg balance and immune homeostasis, we analyzed IL-6 and TGF-β-secreting CD4^+^ T cells. The frequency of IL-6^+^ CD4^+^ T cells significantly increased in the spleens of PbA-infected mice as the parasitemia increased in a day-wise manner and was maximum on 7 DPI (*p* < 0.001). This PbA-mediated increase was reversed in MSC-infused mice as the population of IL-6^+^ CD4^+^ T cells decreased on 3 DPI (*p* < 0.001) and 7 DPI (*p* < 0.001) as compared to PbA-infected mice (Fig. 6C, F). We also checked the mRNA expression levels of IL-6 in spleenocytes and found significant upregulation of IL-6 gene expression in PbA-infected mice (*p* < 0.001), which was again downregulated on account of MSC infusion in infected mice (*p* < 0.001, Fig. 6E). MSC-mediated downregulation in IL-6 level was again confirmed on biochemical analysis of serum, as the level of IL-6 cytokine was significantly lower in the serum of MSC-infused mice as compared to that of PbA-infected mice (Fig. 6G). We also analyzed the TGF-β-secreting CD4^+^ T cells and found an increase in their population, which, however, was not significant (*p* > 0.05, Fig. 6B). We further analyzed the effect of MSC-mediated IL-6 downregulation on Th17/Treg balance, as a strong IL-6 microenvironment shifts the axis of development towards Th17 cells. It was found that PbA infection caused a significant increase in the Th17/Treg ratio from 3 DPI (p < 0.001) to 7 DPI (p < 0.001), representing a bias towards the Th17 response. This skewed balance was reversed by MSCs as they brought the Th17/Treg balance back to homeostatic levels (Fig. 6D). These results indicate that MSCs effectively restored the balance between Th17 and Treg cells by modulating the levels of IL-6 cytokine in PbA-infected mice.

### MSCs induce a novel CD4^+^ T cell subset expressing both RORγt^+^ and Foxp3 transcription factor in PbA-infected mice

**Fig. 7:**
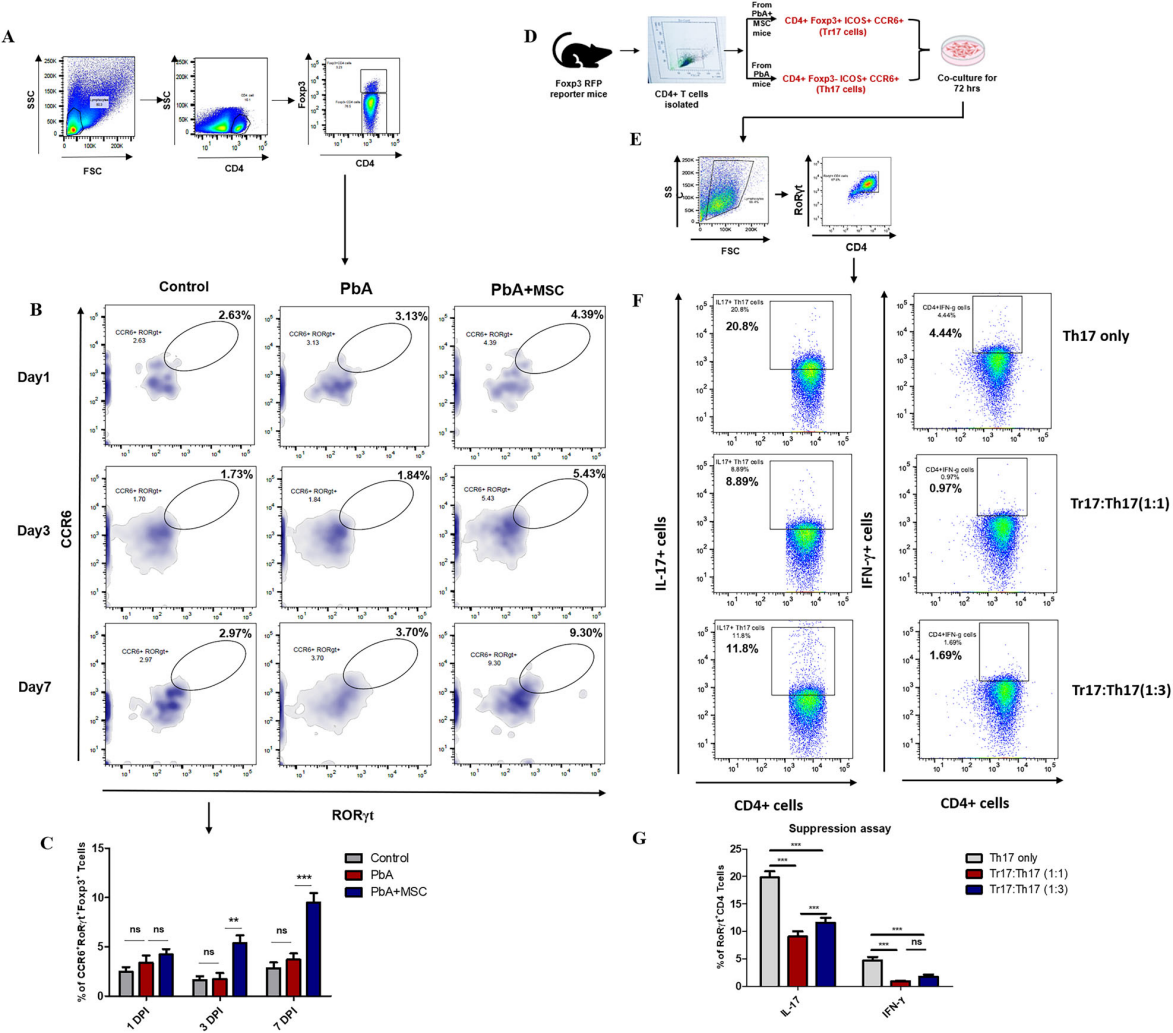
MSCs induce a dual phenotype novel Treg subset expressing both RORγt^+^ and Foxp3^+^ in PbA-infected mice. PbA-infected mice were infused with MSCs, and splenocytes were isolated for characterization of RORγt^+^ Foxp3^+^ Tr17 cells through flow cytometry. **A** Representative FACS plot showing the gating strategy for the identification of Tr17 cells. **B** Representative flow plots and **C** bar graphs showing frequency of CCR6^+^ RORγt^+^ Foxp3^+^ Tr17 cells in spleen of control, PbA-infected, and MSC-infused PbA-infected mice (*n* = 3) on 1, 3, and 7 DPI, respectively. The data shown is representative of two independent experiments. **D** Flow chart showing sorting of cells from Foxp3^RFP^ reporter mice and IL-17 suppression assay. **E** Representative FACS plots showing the gating strategy for identification of Th17 cells. **F** Representative flow plots and **G** bar graphs showing frequency of IL-17^+^ and IFN-γ^+^ Th17 cells in in vitro culture of Th17 only, Tr17:Th17 (1:1), and Tr17:Th17 (1:3) (*n* = 3), respectively. The data shown is representative of two independent experiments. Data are expressed as mean ± SEM. Two-way ANOVA, followed by post hoc Bonferroni method, was used to determine statistical significance. ^*^p < 0.05, ^**^p < 0.01, ^***^p < 0.001, ns non-significant.

Dual phenotype RORγt^+^ Foxp3^+^ CD4^+^ T cells, namely Tr17 cells represent a novel subset of peripheral Treg cells that have high suppressive activity against the antigen-specific Th17 response and are highly associated with CCR6 surface expression [33]. Hence, we checked the effect of MSCs on the population of Tr17 cells in spleen and lymph nodes and found that there was a steady increase in the frequency of RORγt^+^ CCR6^+^ Foxp3^+^ CD4^+^ T cells in splenocytes of MSC infused mice as compared to the PbA infected mice on 3 DPI (*p* < 0.01) and 7 DPI (*p* < 0.001) (Fig. 7B, C). The population of RORγt^+^ CCR6^+^ Foxp3^+^ CD4^+^ T cells in PbA-infected mice was slightly higher than that seen in control mice, suggesting that PbA infection could induce Tr17 cells, but the population was not sufficient to mount an effective response against the activated Th17 cells. To further characterize these cells, we analyzed the expression of Treg-associated molecules on the surface of RORγt^+^ CCR6^+^ Foxp3^+^ CD4^+^ T cells. We found that MSCs increased the population of Tr17 cells that expressed CD25, a Treg cell activation marker (Supplementary Fig. 1C). We also observed an increase in RORγt^+^ CCR6^+^ Foxp3^+^ CD4^+^ T cells expressing CTLA-4, a surface molecule associated with functional aspects of Treg cells. Since most of the Tr17 cells are associated with ICOS^hi^ expression on their surface, we found that MSCs were increasing the ICOS-expressing Tr17 cell population in the spleen of MSC-treated mice as compared to PbA-infected mice (Supplementary Fig. 1C). Collectively, these results suggest that MSCs induce a novel Treg subset while increasing its activation, as well as suppressive activity.

To further check the suppressive activity of these Tr17 cells against the pathogenic Th17 cells, CD4^+^ Foxp3^+^ ICOS^hi^ CCR6^+^ Tr17 cells were sorted from the spleens of MSC-infused PbA-infected Foxp3^RFP^ reporter mice and co-cultured for 72 h with CD4^+^ Foxp3^-^ ICOS^hi^ CCR6^+^ Th17 cells sorted from the spleens of PbA-infected Foxp3^RFP^ reporter mice (Fig. 7D). The CD4^+^ Foxp3^+^ ICOS^hi^ CCR6^+^ Tr17 cells significantly suppressed the activity of pathogenic CD4^+^ Foxp3^−^ ICOS^hi^ CCR6^+^ Th17 cells, as the co-culture of these cells significantly decreased the expression of IL-17 in RoRγt^+^ CD4 + T cells. This suppression was dependent on the Tr17 to Th17 ratio, as the suppression was much higher when the cells were cultured in a 1:1 ratio as compared to when cultured in a 1:3 ratio (Fig. 7F, G). We also checked the expression of IFN-γ in RoRγt^+^ CD4 + T cells, which is secreted by highly pathogenic Th17 cells, and found that similar suppression in the expression of IFN-γ was seen as in the case of IL-17 (Fig. 7F, G). Our results indicate high suppressive capability of Tr17 cells as compared to Treg cells, as the suppression of IL-17 was much higher in the case of co-culture of pathogenic Th17 cells with Tr17 cells, as compared to when co-cultured with Treg cells (Fig. 4F, G).

## Discussion

Conventional therapies for CM fail to address neurological and immunopathological complications, leading to severe organ damage and high mortality. These complications involve dysregulated pro- and anti-inflammatory responses, primarily a balance in the Th17/Treg axis [34], thus necessitating the need for immunomodulatory approaches to manage the disease. In the present study, we evaluate the therapeutic potential of MSCs due to their immunomodulatory properties and demonstrate that MSCs confer significant protection against the development and progression of ECM in PbA-infected C57BL/6 mice. Consistent with previous studies showing rapid parasitemia progression and high mortality in PbA-infected mice [35, 36], our model exhibited nearly 100% mortality by day 9 post-infection. Remarkably, MSC-treated mice displayed a slower parasitemia progression and significantly improved survival rates, highlighting the therapeutic potential of MSCs in ECM. These findings align with reports that MSCs possess anti-parasitic properties and immunomodulatory effects in infectious disease models [37, 38]. A key hallmark of CM pathology is the disruption of the BBB, contributing to neurological deficits [3, 39]. Using Evans blue dye extravasation, we confirmed extensive BBB leakage in PbA-infected mice, which was substantially attenuated following MSC treatment. This protective effect on the BBB is consistent with previous work showing MSCs promote vascular stability and repair through paracrine mechanisms, including the secretion of angiogenic and anti-inflammatory factors [40, 41].

Apoptosis in lymphocytes is a common phenomenon that parasites may exploit to evade host immune responses. In this study, we observed that mesenchymal stem cells (MSCs) reduced the proportion of apoptotic and dead lymphocytes in the spleen, indicating a potential protective role in preventing lymphocyte exhaustion. This aligns with previous findings showing that MSCs can mitigate activation-induced cell death (AICD) in T cells by downregulating the Fas/FasL pathway [42]. Furthermore, MSC treatment led to a decrease in PD-1 expression and reactive oxygen species (ROS) levels in lymphocytes, consistent with our earlier observations [43, 44], further supporting their immunomodulatory and protective functions.

Another major finding of our study is the suppression of Th17-mediated inflammatory responses by MSCs. Th17 cells, characterized by RORγt expression and IL-17 production, have been implicated in the immunopathology of ECM [45, 46]. We observed a significant expansion of Th17 cells during PbA infection, correlating with disease severity, which was effectively diminished by MSC infusion. This reduction in Th17 cells was paralleled by a downregulation of IL-17 mRNA expression and lower serum IL-17 cytokine levels, suggesting that MSCs dampen pathogenic Th17 responses during ECM. Interestingly, we found that MSCs promoted an increase in regulatory T cells (Tregs), particularly Foxp3^+^ CD4^+^ T cells, and increased their suppressive activity by upregulating CTLA-4 molecules on their surface, which are known to suppress effector T cells [47]. Tregs are crucial for controlling excessive inflammation and maintaining immune tolerance [48], and their role in mitigating CM has been previously highlighted [48]. Our data show that MSC therapy enhances Treg function, evidenced by upregulated IL-10 production, both at the transcript and protein levels, consistent with the known ability of MSCs to induce Treg differentiation and IL-10 secretion [49]. MSCs also upregulate ICOS on Tregs, which are known to produce high IL-10 levels [50, 51], which is further known to suppress Th17-mediated inflammatory response [52].

We further demonstrate that MSCs restore the disrupted Th17/Treg balance during PbA infection. The Th17/Treg differentiation axis is critically regulated by cytokines such as IL-6 and TGF-β [53]. Our results show that MSC treatment leads to a marked decrease in IL-6 levels without significantly altering TGF-β [54], favoring Treg differentiation and suppressing Th17 expansion. Although MSCs are known to produce some level of IL-6 but this amount is very low as that produced by T cells, and they are often known to suppress IL-6 production from antigen-presenting cells and T-cells, thus creating a more tolerogenic environment [55, 56]. MSCs are also known to regulate Th17/Treg balance through the secretion of certain soluble factors like NO and PGE2, which have been reported to enhance Treg response and dampen Th17 response [49, 57]. MSCs isolated from the spleens of PbA-infected mice produced copious amounts of NO and PGE2 in vitro as compared to the MSCs isolated from bone marrow. This differential secretion of nitric oxide (NO) and prostaglandin E2 (PGE2) by MSCs might be explained due to the inflammatory microenvironment in the spleens of PbA-infected animals expressing high levels of IFN-γ, TNF-α, and IL-1β (data not shown), which enhances the immunosuppressive nature of the MSCs [55]. Inhibition of COX2 (responsible for PGE2 secretion) and iNOS (responsible for NO secretion) in PbA-infected mice by Celebrex, which is known to inhibit COX2 directly and iNOS through NFκB and JNK pathway [57], increased parasitemia and decreased survival of the animals to some extent (Supplementary Fig. 1F–I), suggesting their possible role in influencing disease immunopathogenesis.

For the first time, our study reports the identification of an increased population of RORγt+Foxp3+ regulatory T cells, known as Tr17 cells, in the spleens of MSC-treated mice. These cells, characterized by dual expression of Th17 markers along with Treg markers, have high CCR6 surface expression [58] and potent suppressive activity against Th17 responses [14, 15]. MSC therapy not only expanded the Tr17 subset but also enhanced their activation status, as shown by increased expression of CD25 and ICOS, along with increased suppressive activity, as shown by increased CTLA-4 expression. Our study also confirms their high suppressive activity against Th17 cells as in vitro co-culturing of these cells with pathogenic Th17 cells, suppressed the activity of Th17 cells by downregulating both IL-17, as well as IFN-γ expression in RORγt^+^ CD4 + T cells and this suppression was comparatively higher (2–2.5 fold decrease in IL-17) as compared to the suppression seen when Th17 cells were co-cultured with Treg cells (1.43–1.67 fold decrease in IL-17). This suggests that MSCs may promote a unique regulatory subset that are highly suppressive towards pathogenic Th17 cells and further contributes to disease amelioration. A cytokine milieu rich in IL-6, IL-1β, and TGF-β promotes Stat3-dependent RORγt induction and Th17 differentiation, while Treg responses and their plasticity toward Tr17-like (RORγt⁺Foxp3⁺) states are shaped by the balance and timing of these signals [59]. Kim et al. [14] have shown the involvement of both IL-10 and IL-6-mediated induction of RORγt and CCR6 via Stat3 for the development of Tr17 [14]. However, in our study, MSCs led to a decrease in IL-6 levels and an increase in IL-10 levels. Thus, IL-10 cytokine might play a major role in the induction of Tr17 cells in MSC-treated mice. Yang et al. [59] reported an elevation in IL-23 and IL-23R along with an increase in the population of RORγt⁺Foxp3⁺ T cells [60]. However, there has been no link established between IL-23 and the induction of RORγt⁺Foxp3⁺ T cells. Similarly, there is no report linking IL-1β and Tr17 induction, and further studies are warranted to establish the role of IL-23 and IL-1β in Tr17 induction.

In human cerebral malaria (HCM), an imbalance between Th17 and regulatory T (Treg) cells has been associated with disease severity. Clinical studies have shown increased frequencies of IL-17-producing CD4⁺ T cells during acute infection, along with altered or functionally impaired Foxp3⁺ Tregs, suggesting dysregulated immune homeostasis [61]. This balance likely determines whether inflammation contributes to CNS pathology or is mitigated by effective regulatory activity. There are no current studies regarding Tr17 in malaria. Thus, to fully understand the role of MSCs in modulating Th17/Treg dynamics from a translational perspective, the current findings need to be extrapolated and validated in HCM cases. However, that is difficult due to limited CNS and longitudinal immune profiling in human subjects, which makes the precise contribution of Th17/Treg imbalance to CM pathogenesis unpredictable in clinical cases.

Overall, our study highlights the multifaceted immunomodulatory roles of MSCs during ECM: reducing Parasitemia, preserving BBB integrity, suppressing pathogenic Th17 responses, enhancing Treg-mediated regulation, restoring immune balance, and inducing novel regulatory subsets that show high suppressive activity against pathogenic Th17 cells. Further, these findings provide strong support for the use of MSC-based therapies as a novel adjunct treatment for CM.

Despite mesenchymal stem cells (MSCs) showing significant therapeutic promise, several limitations remain. First, variability in MSC sourcing, such as donor-to-donor differences, tissue of origin, and culture conditions, can affect their immunomodulatory and regenerative properties [62]. Second, standardization of dosing and delivery routes with optimal cell numbers, timing, and administration methods is still not well defined [63]. Additionally, long-term safety and efficacy data are limited, and concerns such as potential for unwanted differentiation, reduced potency after prolonged expansion in vitro, and heterogeneity between batches complicate their clinical translation [54]. These factors underscore the need for rigorous standardization and controlled clinical studies to fully establish the therapeutic potential of MSCs. Scientist are working in this direction to enhance their therapeutic potential by using different drugs, biomolecules, and biomaterials.

## Conclusion

In summary, our study provides strong evidence that MSC therapy offers a multifaceted protective effect against experimental CM. MSC treatment reduced parasitemia, improved survival, preserved BBB integrity, and modulated immune responses by suppressing pathogenic Th17 cells while enhancing regulatory T cell populations, including the emergence of RORγt^+^ Foxp3^+^ Tr17 cells. By restoring the critical Th17/Treg balance, MSCs effectively curtailed inflammatory damage and promoted immune tolerance. These findings highlight the therapeutic potential of MSCs as an adjunctive strategy for the management of CM. Further mechanistic studies and clinical translation are warranted to fully harness MSC-based therapies in malaria-endemic settings.

## Supplementary information

Supplementary Figure

Supplementary Figure Legend

## Data Availability

All data generated or analyzed during this study are included in this published article and its supplementary information and are available from the corresponding author on reasonable request.
