# Supplementary figures and images for: Mesenchymal stem cells alleviate experimental cerebral malaria disease severity by inducing RoRγt+ Foxp3+ T regulatory (Tr 17) cells and modulating the dysregulated Th17/Treg axis
Source: Cell Death Discov. 2026 Jan 30;12:87. doi: 10.1038/s41420-025-02900-3 (PMC12876865; doi:10.1038/s41420-025-02900-3)

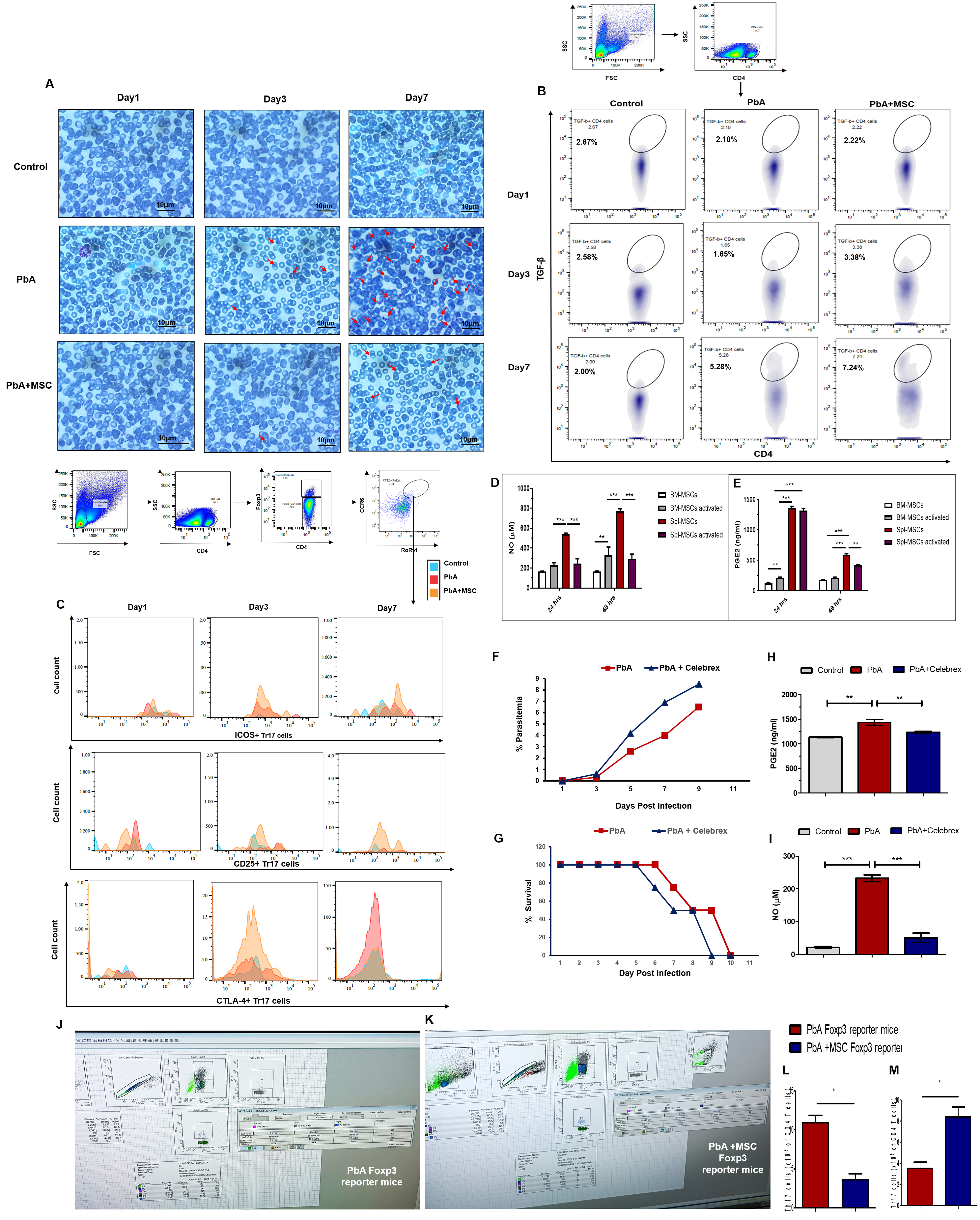

Supplement: Supplementary file 1 — Supplementary Figure [file 41420_2025_2900_MOESM1_ESM.tif]
