# Supplementary material for: Mesenchymal stem cells alleviate experimental cerebral malaria disease severity by inducing RoRγt+ Foxp3+ T regulatory (Tr 17) cells and modulating the dysregulated Th17/Treg axis
Source: Cell Death Discov. 2026 Jan 30;12:87. doi: 10.1038/s41420-025-02900-3 (PMC12876865; doi:10.1038/s41420-025-02900-3)
Supplement: Supplementary file 2 — Supplementary Figure Legend [file 41420_2025_2900_MOESM2_ESM.docx]

**Fig. Supplementary1.** **(A)** Microphotographs showing parasite load in thin blood smears, **(B)** Flow plots showing TGF-β CD4^+^ T cells **(C)** Histograms showing ICOS, CD25 and CTLA-4 expression on Tr17 cells. Tracking of CFSE labelled MSCs after 24 hrs. of infusion. Bar graphs showing MSC secreted NO levels **(D)** and PGE2 levels **(E),** Parasitemia, survival **(F, G)**, PGE2 and NO levels **(H, I)** in serum of PbA and PbA+Celebrex (n=4) mice **(J,K)** sorting of cells from PbA and PbA+MSC infused Foxp3^RFP^ reporter mice, **(L, M)** Total sorted Th17 and Tr17 cells.
